# Identification of Patients with Reinke’s Edema Through Biomechanical Voice Analysis

**DOI:** 10.3390/jpm16030133

**Published:** 2026-02-28

**Authors:** Isabel Cardoso López, Walter Orlando Tenesaca Pintado, Ángel Rodríguez Paramás, Roberto Fernández-Baillo Gallego de la Sacristana

**Affiliations:** 1 Department of Otorhinolaryngology, Vithas Arturo Soria University Hospital, European University of Madrid, Calle Arturo Soria, 103, 28043 Madrid, Spain; cardosis@vithas.es (I.C.L.);; 2 Department of Otorhinolaryngology, University Hospital of Guadalajara, University of Alcalá, 19002 Guadalajara, Spain; 3 Department of Surgery, Medical Sciences and Health, University of Alcalá, 28805 Alcalá de Henares, Spain

**Keywords:** laryngeal cancer, Reinke’s edema, voice pathology, biomechanical analysis, mucosal wave, laryngeal anatomy

## Abstract

**Background and objectives**: Reinke’s edema is a benign disease of the vocal folds caused by smoking and excessive vocal effort that usually leads to chronic dysphonia, especially in women. Diagnosis requires direct evaluation of the vocal folds using videolaryngoscopy. Biomechanical analysis of the voice makes it possible to obtain from a sound sample a set of parameters that describe the pattern of voice production associated with the specific architecture of each vocal fold. The objective is to identify the characteristic vocal production pattern in Reinke’s edema while analyzing the validity of this methodology for screening the pathology. **Methods**: The study was performed with a sample of 175 women, from 26 to 74 years old, separated into 3 groups: Control Group, 52 participants; Reinke’s edema Group, 26 patients; Vocal Fold Pathology—no Reinke’s edema, 97 patients. All the patients were evaluated by the biomechanical analysis tool App Online Lab VCS^®^ by Voice Clinical Systems^®^. **Results**: It is observed that a decrease in F0 (Pr01), an increase in the mucosal wave in the opening phase (Pr20), a shortened closure phase (Pr04) and the presence of mass effect (Pr22) are the main features that characterize Reinke’s edema compared to the control group with broad statistical significance (*p* < 0.001). These results establish that the screening based on the joint presence of the decrease in F0 (Pr01) and the increase in the mucosal wave effect (Pr18/Pr20) presents high sensitivity and specificity indices: Group control vs. Reinke’s edema, Specificity: 0.92, Sensitivity: 0.77; Reinke’s edema vs. Vocal Fold Pathology—no Reinke’s edema, Specificity: 0.93, Sensitivity: 0.77. **Conclusions:** Biomechanical voice analysis objectively identifies voice patterns in women with Reinke’s edema, aiding in effective screening.

## 1. Introduction

Reinke’s edema is a chronic inflammatory condition characterized by the accumulation—usually bilateral—of fluid in the superficial layer of the lamina propria, known as Reinke’s space, in the membranous portion of the vocal folds. It was first described by Hajek in 1891 [1].

Although other risk factors such as vocal abuse or laryngopharyngeal reflux [2] have been implicated, the primary cause is tobacco use [3], which induces hypoxia and leads to the release of endothelial growth factors. Over time, this results in increased vascularization of the lamina propria due to the proliferation of dilated and fragile capillaries, facilitating plasma extravasation into Reinke’s space [4], causing thickening of the vocal folds. This thickening results in a vocal pitch lower than expected for the patient’s age and gender.

Reinke’s edema accounts for between 4% and 16% of laryngeal surgeries [5] and is more frequently diagnosed in women than in men [6]. This difference is attributed not to a higher incidence in women but to the fact that a deepened voice is more often a reason for medical consultation among women [7].

**Figure 1:**
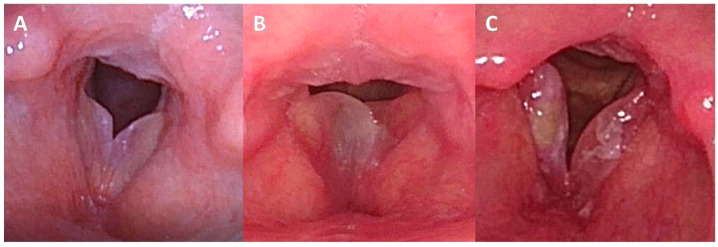
Reinke’s edema observed via distal-chip flexible laryngoscopy. (**A**) Mild-to-moderate, symmetric Reinke’s edema. Pr01: 172.9 Hz; Pr20: 200 r.u. (**B**) Severe, asymmetric Reinke’s edema with predominant involvement of the right vocal fold. Pr01: 128 Hz; Pr20: 800 r.u. (**C**) Moderate Reinke’s edema with extensive leukoplakia on the right vocal fold, resulting in mucosal wave limitation (N-69). Pr01: 198.6 Hz; Pr20: 0 r.u. The mucosal wave limitation caused by the leukoplakia counteracts the typically increased mucosal wave associated with Reinke’s edema.

Diagnosis of Reinke’s edema is based on visual examination of the vocal folds, which appear thickened and fluctuant. With significant volume, the folds may overlap during phonation and lose contact between their free edges (Figure 1). Under stroboscopic light, an increased amplitude of the mucosal wave can be observed due to the flaccidity of the folds. Acoustic analysis of the voice signal typically shows a reduced fundamental frequency, while distortion parameters (Shimmer, Jitter, HNR) may remain within normal limits and show no characteristic pattern [8].

Treatment of Reinke’s edema involves eliminating risk factors, correcting improper vocal behavior through voice therapy, and removing redundant tissue via laryngeal microsurgery. Biomechanical voice analysis examines the mechanical and structural factors involved in the movement of the free edge of the vocal folds, using models based on the underlying histological structure. This study evaluates the validity of biomechanical voice analysis in identifying Reinke’s edema and characterizing the mechanisms that contribute to its development, persistence, or onset.

Beyond these aims, the study also seeks to determine whether biomechanical markers can provide objective indicators of tissue alteration that correlate with clinical findings and endoscopic severity. Since Reinke’s edema is fundamentally associated with changes in the viscoelastic properties of the superficial lamina propria, a method capable of quantifying vibratory irregularities and mass-related alterations may offer additional diagnostic value and support clinical decision-making. Furthermore, by analyzing how the mechanical behavior of the vocal folds deviates from normative patterns, biomechanical voice analysis may clarify the extent to which edema, increased mass, or impaired mucosal wave propagation contribute to dysphonia. Establishing these relationships could improve early detection, help differentiate Reinke’s edema from other mass-loading conditions, and provide clinicians with objective tools to monitor treatment outcomes or disease progression.

## 2. Materials and Methods

The study was designed as an analytical cross-sectional observational investigation aimed at evaluating the screening capability of the proposed method for the detection of Reinke’s edema. The primary sample included 175 women treated in the Department of Otorhinolaryngology, Vithas Arturo Soria University Hospital and classified into three groups: controls without vocal pathology (GC, *n* = 52), patients with Reinke’s edema (REG, *n* = 26), and patients with other vocal pathologies (NER, *n* = 97). All participants provided informed consent.

Voice samples from all participants in the study were consistently collected by the same otorhinolaryngology specialist in a consultation room without ambient noise, using a Samsung Galaxy S8 mobile device (Electronics Co., Ltd. Suwon, South Korea) equipped with an external Saramonic SmartMic Professional TRRS condenser microphone (Saramonic, North White Plains, New York, United States). The recorded sample consisted of a non-nasalized /a/phonation at a natural pitch for 4 s, captured at approximately 15 cm from the participant’s mouth [9].

Visual examinations of the OC participants were performed and recorded using an Olympus Visera Elite OTV-S190 flexible videolaryngoscope (Olympus Corporation, Hachioji, Tokyo, Japan) equipped with a chip-on-tip digital camera, which provided high-resolution imaging of the laryngeal structures. A stroboscopic light source (Strobolux III, Optomic) was employed to enable detailed assessment of vibratory function during phonation. All recordings were captured and stored using a Game Capture HD II device (AVerMedia Technologies, Inc. New Taipei City, Taiwan), ensuring stable acquisition and high-quality playback for subsequent analysis.

For all studied cases, signal processing for the extraction of biomechanical parameters associated with voice production was performed using the OnlineLab VCS^®^ Android version developed by Voice Clinical Systems^®^ [10,11,12,13,14,15,16], and a comprehensive R3 biomechanical report (version 9.1.6.2024) was generated via the virtual lab (see Table 1).

A statistical study was conducted using SPSS version 25 to assess the significance of the mean differences between groups. To do this, the ANOVA test was applied, allowing variables with statistically significant differences between the groups analyzed to be identified. Subsequently, a principal components analysis (PCA) was performed to reduce the dimensionality of the data and facilitate the grouping of cases based on the observed patterns. This procedure allowed the identification of the most relevant parameters explaining the variability between cases. Based on the most significant parameters obtained from the PCA and ANOVA, a decision-making algorithm was established for the screening of pathological cases. This algorithm was evaluated in terms of its diagnostic capacity, assessing its sensitivity and specificity to effectively discriminate between normal and pathological cases.

## 3. Results

### 3.1. Descriptive Analysis

The sample was divided into three groups, obtaining the following results in the descriptive study:

- Control Group (CG): This group included 52 women with no clinical signs of dysphonia in the previous 3 weeks (to avoid residual acute laryngeal pathology that could affect biomechanical analysis) and no organic lesions on videolaryngoscopy. Ages ranged from 38 to 60 years (mean age: 46.88 years).
- Reinke’s Edema Group (REG): This group included 26 women with videolaryngscopic diagnosis of Reinke’s edema, those aged between 40 and 74 years (mean age: 59.88 years).
- Pathology Group (PG): This group included 97 women with videolaryngoscopic diagnosis of vocal pathology other than Reinke’s edema, aged 26 to 74 years (mean age: 48.78 years). The categorization of pathologies was as follows: unilateral vocal fold paralysis, 4.1%; superficial leukoplakia, 4.1%; sulcus vocalis, 8; vocal cyst, 1%; vocal polyp, 10.3%; vocal nodules, 33%; acute laryngitis, 18.6%; presbyphonia, 7.2%; adductor spasmodic dysphonia, 1%; hyperfunctional dysphonia, 2.1%; hypofunctional dysphonia, 10.3%.

### 3.2. Results Obtained from the Biomechanical Analysis

#### 3.2.1. Fundamental Frequency (Pr01)

Comparative analysis of fundamental frequency (F0) between the control group and patients with Reinke’s edema showed a significant reduction in F0 in the latter (*p* < 0.001). Specifically, 71.8% of patients had F0 values below 160 Hz, indicative of free edge thickening, compared to 3.8% observed in the control group.

#### 3.2.2. Vocal Cycle Phase Durations (Pr04–Pr05)

A significant reduction in closed phase duration, within pathological range, was observed in the Reinke’s edema group compared to controls (*p* < 0.001). Despite the thickness of the free edge, contact between the vocal folds becomes less effective, prolonging the opening phase (Pr05) and causing a loss of energy that reduces glottal efficiency. The differences in the duration of this phase are statistically significant, with an average increase of 27% in the group with REG compared to the CG.

#### 3.2.3. Vocal Effort and Strain Parameters (Pr08–Pr09)

Parameter Pr08, representing vocal strain during glottic closure, was significantly elevated in the Reinke’s edema group (*p* < 0.001), as was Pr09, which reflects force at the free edge during closure (*p* < 0.001). These results show that patients with Reinke’s edema must make a greater compensatory effort to counteract the mass effect produced by the thickening of the free edge and achieve effective glottic closure that allows the desired F0 to be reached. In the group with Reinke’s edema (REG), the tension (Pr08) increases, on average, by 338% (CG = 9.7 u.r.; REG = 42.5 u.r.), while glottic squeeze (Pr09) increases by 219% (CG = 562.3 u.r.; REG = 1785.5 u.r.).

#### 3.2.4. Glottic Efficiency (Pr10)

Average glottal efficiency was lower in patients with Reinke’s edema than in the control group, constituting a clinical finding in 88.95% of cases. However, the differences between the two groups were not statistically significant (*p* > 0.005).

#### 3.2.5. Mucosal Wave (MW) in the Closed Phase (Pr17–Pr19)

In Reinke’s edema patients, the MW adequacy index tended to remain within the normal range, while in the control group, it was slightly reduced. However, no significant difference was found (*p* > 0.005).

#### 3.2.6. Mucosal Wave (MW) in the Opening Phase (Pr 18–Pr20)

**Figure 2:**
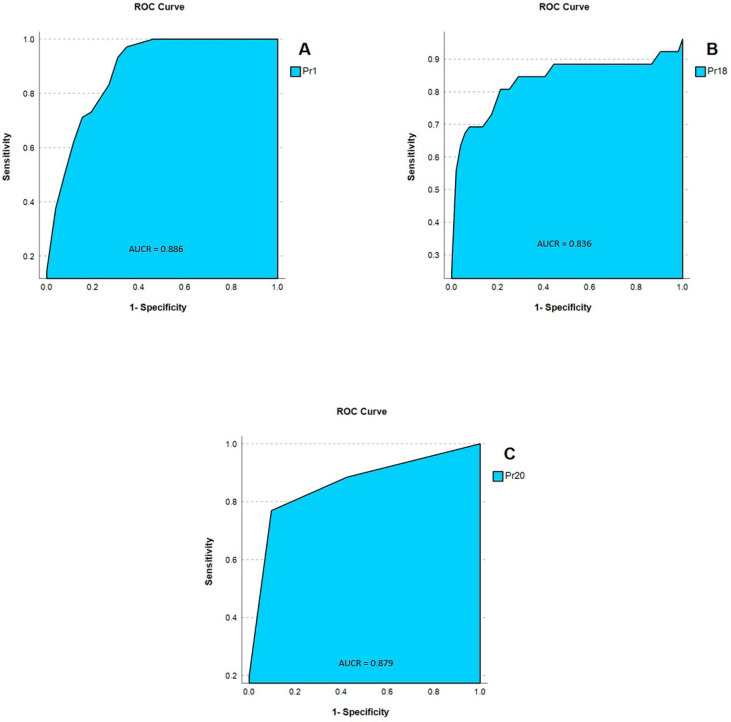
The ROC curves corresponding to the three parameters with the greatest discriminatory capacity for differentiating Reinke’s oedema from the normal population are presented: (**A**) Pr1, Fundamental Frequency; (**B**) Pr18, Mucosal wave correlation in the closing phase; (**C**) Pr20 Correlation of oedema during the mucosal wave in the open phase.

The Reinke’s Edema group showed a marked increase in MW amplitude during the opening phase, affecting 90% of patients, compared to controls (*p* < 0.001) (Figure 2).

#### 3.2.7. Glottic Impact Parameters (Pr21–Pr22)

In the group with Reinke’s edema, there was a marked alteration of the free edge, reflected in elevated mass effect values (Pr22), which were significantly higher than those of the control group (*p* < 0.001). However, no significant differences were found in the mass effect of the free edge when comparing the group with Reinke’s edema with the group with other vocal pathologies (*p* > 0.001). This pattern suggests that Reinke’s edema has distinctive acoustic properties that do not depend solely on the increase in mass at the free edge but rather respond to pathophysiological characteristics specific to this entity.

### 3.3. Screening Reinke’s Edema via Biomechanical Analysis

Using biomechanical parameters, combining a decrease in F0 below normal female range (Pr01 < 180 Hz) with increased MW indices (Pr18 ≥ 90 u.r. and Pr20 ≥ 200 u.r.), the analysis correctly identified 76.9% of Reinke’s edema cases compared to controls (Table 2).

This screening method was applied to a broader group of 123 patients with various vocal pathologies, including the 26 patients diagnosed with Reinke’s edema (*RE*). The analysis identified 77% of Reinke’s edema cases as positive and correctly excluded 93% of non-Reinke’s pathologies (Table 3).

## 4. Discussion

Voice results from airflow acting upon the vocal folds [18]. The MW—and therefore the vocal signal—depends on airflow and the biomechanical properties of the vocal folds. The vocal fold structure is described as a layered system [19]. Histologically, it contains three layers: epithelial mucosa, lamina propria (with superficial, intermediate, and deep divisions—Reinke’s space included), and the vocal muscle. The viscoelastic structure of the lamina propria, determined by fiber density and composition, defines its biomechanical behavior [20]. Biomechanically, the folds are divided into three functional layers: Cover (epithelial mucosa + superficial lamina propria); Transition (intermediate + deep lamina propria -vocal ligament-; and Body (vocal muscle).

The lining epithelium must withstand repeated contact between the vocal folds. Reinke’s space, the most vibratory portion of the vocal fold, is rich in extracellular matrix proteins and has few fibers, and its thickness is maximum in the intermediate third of the vocal folds [21]—the primary contact region during phonation. In the transition layer, collagen and elastin fibers allow the ligament to adapt to length and strain changes (anteroposterior and lateromedially), modulating F0 and ensuring proper fold contact [22]. The vocalis muscle (thyroarytenoid) regulates vocal fold stiffness and strain [23], while the cricothyroid muscle—external to the fold—modulates elongation, increasing F0. Thus, the structure determines the biomechanical signal, and any pathological disruption alters voice production.

Pathophysiology of Reinke’s Edema. Histologically, Reinke’s edema is characterized by increased subepithelial vascularization [4] due to dilated and fragile capillaries. Plasma extravasation occurs during vibration through endothelial pores. Inflammatory cells expressing VEGF are also present, responding to hypoxia from smoking or phonation [24]. The increased volume in Reinke’s space disrupts vocal fold architecture, limiting pitch modulation despite functioning transition and body layers. The larger the edema, the greater the impairment. Diagnosis is based on visual assessment (videolaryngoscopy), allowing for severity grading [25,26]. This study included only female patients, due to the clear gender predominance (13:1) in our ENT department; and consistent findings in the literature [7], but it should be noted that the findings observed in this study cannot be automatically extrapolated to the male population.

**Figure 3:**
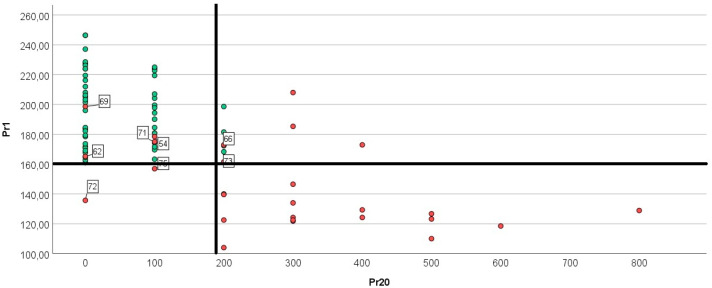
Scatterplot of cases from CG (green circles) versus cases from REG (red circles), based on F0 values (Pr01) and the mucosal wave correlate during opening (Pr20). Cases located below the horizontal dotted line (F0: 160 Hz) exhibit severely reduced F0, while the vertical line (200 r.u.) marks the upper limit for severe Pr20 values (200 r.u.). The plot shows that most Reinke’s edema cases cluster around Pr01 values below 160 Hz and Pr20 values above 200 r.u. Outliers from this distribution include: (1) cases with very mild, compensable edema or coexisting pathology that counteracts the edema (e.g., N-69, see Figure 1), which are grouped in the upper left quadrant; (2) cases with increased mucosal wave during closure but not during opening (e.g., N-62); and (3) cases where the vocal biomechanics suggest difficulty in generating sufficient subglottic pressure to initiate a mucosal wave—such as the presence of a glottic gap (N-72) or severely prolonged open phase duration (N-75).

Biomechanical Analysis in Reinke’s Edema. Acoustic voice analysis shows F0 reduction proportional to edema size, but parameters like jitter, shimmer, or Noise-to-Harmonics Ratio do not define a characteristic pattern [27], although the combination of acoustic, aerodynamic and electroglottographic parameters of a patient with Reinke’s edema is useful to establish a comparison before and after surgical treatment [28]. However, biomechanical voice signal analysis provides correlations of fold dynamics and accurately describes each patient’s vocal production pattern. In Reinke’s edema, the pathological biomechanical voice pattern is characterized by alterations at three levels:

1. Structural Alterations: Marked F0 reduction (Pr01), often within male frequency range. Increased MW amplitude in opening (Pr20), due to redundant flaccid tissue fluttering with subglottic airflow (Figure 3).
2. Functional Consequences: Shortened closed phase duration (Pr04–Pr05) due to floppy consistency of folds, preventing stable contact despite increased volume [29]. Mass effect at the free edge impeding closure, producing glottic impact (Pr21–Pr22).
3. Compensatory Mechanisms: Include increased phonatory effort and heightened muscular tension to achieve and maintain glottal closure (Pr08–Pr09), with the goal of enhancing glottal efficiency and counteracting the drop in fundamental frequency.

Correlate of the mucosal wave. The marked reduction in the closure phase observed in patients with Reinke’s edema limits the possibility of reliably evaluating the expected increase in the mucosal wave associated with the pathophysiology of this entity. The alteration and shortening of this phase prevent biomechanical biomarkers from adequately reflecting the vibratory behavior of the free edge during glottic closure. In contrast, the large and increased movement of the surface edge characteristic of Reinke’s edema is clear during the open phase, where the mucosal wave can be consistently detected and quantified using biomechanical voice biomarkers.

Screening Performance. The combined use of reduced Pr01 and elevated Pr18/Pr20 achieved a sensitivity and specificity of 77% for identifying Reinke’s edema when compared with healthy controls (Figure 2). This indicates that the biomechanical profile defined by these parameters is highly representative of the functional alterations associated with the disorder, particularly the increased mass and impaired vibratory efficiency of the superficial lamina propria. When the same criteria were applied to individuals with other laryngeal pathologies, certain biomechanical patterns showed partial resemblance to those seen in Reinke’s edema. For example, a reduction in fundamental frequency may also occur in acute laryngitis due to inflammatory thickening, while an increased mucosal wave can be observed in lesions such as vocal fold polyps, which also modify the pliability of the free edge. Despite these overlaps, the screening approach demonstrated strong discriminatory capability: it correctly identified 77% of Reinke’s edema cases while maintaining a low false-positive rate of only 7%. This balance between sensitivity and specificity suggests that biomechanical voice analysis may serve as a valuable adjunct tool for early detection, differential diagnosis, and clinical decision-making in patients presenting with mass-related vocal fold alterations (Table 3).

**Figure 4:**
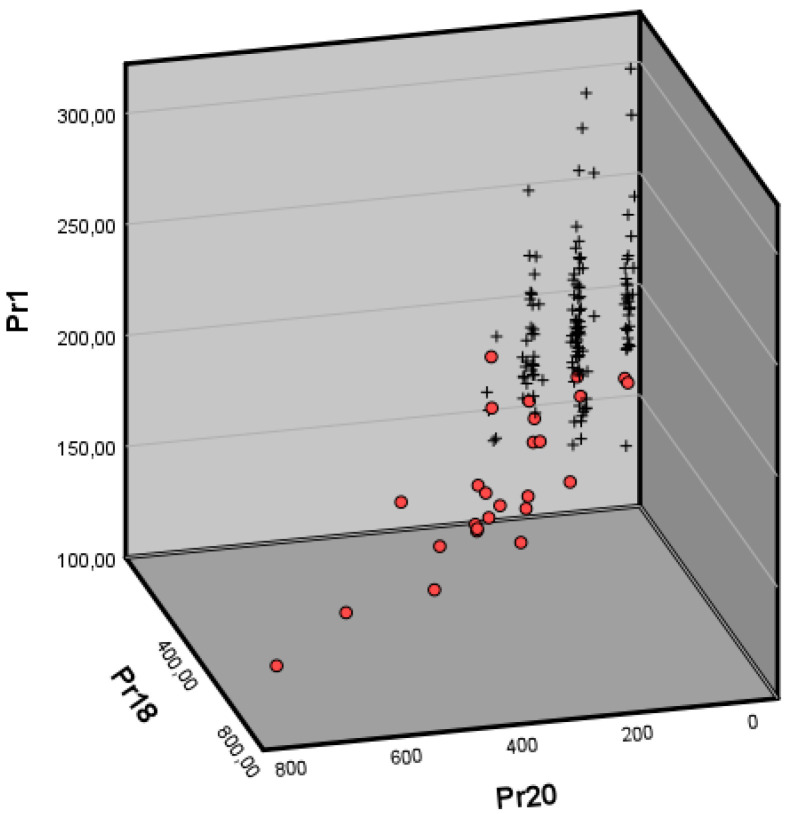
Scatterplot showing the distribution of patients with general voice pathology (crosses) versus those with a main diagnosis of Reinke’s edema (circles), based on the main parameters of the study: F0 (Pr01), mucosal wave correlation in the closing phase (Pr18), and correlation of edema during the mucosal wave in the open phase (Pr20).

Several false positives were likely attributable to excess mucus behaving as a mass during phonation [30], underscoring the importance of adequate vocal clearing before recording. Finally, in a subset of Reinke’s cases, coexisting lesions (Figure 1) modified the biomechanical pattern; however, the screening method still accurately identified the underlying condition (Figure 4). In contrast, most of the false negatives observed in Figure 4 corresponded to mild presentations of Reinke’s edema. In these early stages, the mucosal wave remains largely preserved, and the characteristic vibratory impairment caused by increased mass is not yet pronounced enough to meet the detection threshold of the screening parameters. These milder cases are often accompanied by a glottic gap during phonation; because the free edges do not achieve full contact, the amplitude and propagation of the mucosal wave appear reduced, creating a pattern that can mask the initial biomechanical consequences of edema and thereby reduce sensitivity.

Another compensatory mechanism appears to contribute to false-negative classifications. Some patients actively counteract the typical pitch-lowering produced by thickening of the free edge by increasing their fundamental frequency. They achieve this by elongating the vocal folds and elevating longitudinal tension, which decreases the mucosal wave. This compensatory increase in F0 produces a biomechanical profile that more closely resembles that of normal phonation, allowing mild cases of Reinke’s edema to remain undetected by the screening model.

## 5. Conclusions

Reinke’s edema is a frequent cause of chronic dysphonia, particularly in women. This study demonstrates that biomechanical voice analysis can identify the characteristic pathological vocal production patterns associated with this condition. As a non-invasive, objective, and reproducible method, it offers significant potential as a screening tool for diagnosing Reinke’s edema. Early identification of vocal biomechanical alterations may facilitate timely referral, improve patient outcomes, and contribute to the detection of early-stage laryngeal pathologies. Therefore, integrating biomechanical voice analysis into routine clinical practice as a screening and monitoring tool complementary to videolaryngostroboscopy could enhance diagnostic accuracy and support preventive strategies in voice-related disorders.

## Figures and Tables

**Table 1:** Normal and pathological ranges of biomechanical parameters presented in the study. Abbreviations: (r.u) Relative units; (Hz.) Hertz; (SER) Severe reduction; (SLR) Slight reduction; (N) Normality; (SLE) Slight elevation; (SEE) Severe elevation. Parameters: (Pr01) Fundamental Frequency; (Pr04) Closed phase; (Pr5) Open phase; (Pr08) Strain; (Pr09) Effort; (Pr10) Efficiency; (Pr17) Mucosal wave correlation in the closed phase; (Pr18) Mucosal wave correlation in the opening phase, (Pr19) Correlation of edema in the mucosal wave during the closed phase (Pr20) Correlation of edema in the mucosal wave during the opening phase; (Pr21) Structural imbalance at the free edge of the vocal cords; (Pr22) Correlate of possible mass at the free edge of the vocal cords [17].

	Pr01 (Hz.)	Pr04 (%)	Pr05 (%)	Pr08 (%)	Pr09 (%)	Pr10 (r.u)	Pr17 (r.u)	Pr18 (r.u)	Pr19 (r.u)	Pr20 (r.u)	Pr21 (r.u)	Pr22 (r.u)
(SER)	<160	<50	<25	<0.46	<40	<1	<130	<10	<(−40)	-	-	-
(SLR)	160–179	50–54	25–29	0.46–1	40–80	1–1.2	130–189	10–19	(−40)–(−9)	-	-	-
(N)	180–240	55–70	30–45	1.0–26	80–749	1.2–1.7	190–330	20–65	(−10)–60	0–99	<75	0
(SLE)	241–260	71–75	46–50	27–44	750–1360	1.8–2.3	331–370	66–100	60–90	100–199	75–84	>0
(SEE)	>260	>75	>50	>44	>1360	>2.3	>370	>100	>90	>200	>85	>0
$\bar{X}$ CG	195.1	56.5	43.4	9.7	562.3	0.75	150.2	68.3	−13.6	51.9	70.2	0
$\bar{X}$ REG	145.6	44.4	55.5	42.5	1795.5	0.63	168.0	242.2	6.4	288.4 *	84.3	0.79 **

* Indicative value of the correlate of edema in the vocal cord; ** Mass correlate at the free edge.

**Table 2:** Screening results for the full patient cohort (OC-N Control Group and RE) using the criteria of decreased F0 and increased mucosal wave (Pr18/Pr20). Abbreviations: PPV: Positive Predictive Value; NPV: Negative Predictive Value.

	*CG* (N:52)		*REG* (N:26)	
↓ F0 + ↑ Pr18 ± ↑ Pr20	No	Yes	No	Yes
	48	4	6	20
Sensitivity			0.77	
Specificity	0.92			
PPV			0.83	
NPV	0.89			

**Table 3:** Screening results comparing patients with general voice disorders versus those with a main diagnosis of Reinke’s edema, using the criteria of decreased F0 and increased mucosal wave (Pr18/Pr20) [17].

	*PG* (N:97)		*REG* (N:26)	
↓ F0 + ↑ Pr19 ± ↑ Pr20	No	Yes	No	Yes
	90	7	6	20
Sensitivity			0.77	
Specificity	0.93			
PPV			0.74	
NPV	0.94			

## Data Availability

The raw data supporting the conclusions of this article will be made available by the authors on request.

## Abbreviations

The following abbreviations are used in this manuscript:

CG	Non-pathological cases
REG	Cases of Reinke’s edema
PG	Vocal Fold Pathology—no Reinke’s edema
MW	Mucosal Wave
r.u	Relative units
Hz	Hertzs.
SER	Severe reduction
SLR	Slight reduction
N	Normality
SLE	Slight elevation
SEE	Severe elevation
Pr01	Fundamental Frequency
Pr04	Closed phase
Pr05	Open phase
Pr08	Strain
Pr09	Effort
Pr18	Mucosal wave correlation in the closing phase
Pr20	Correlation of edema during the mucosal wave in the open phase
Pr21	Structural imbalance at the free edge of the vocal cords
Pr22	Correlate of possible mass at the free edge of the vocal cords
PPV	Positive Predictive Value
NPV	Negative Predictive Value
AUR	Area under ROC curve
$\bar{X}$ CG	Average value in the control group
$\bar{X}$ REG	Average value in the group of patients with Reinke’s edema
